# Feasibility of Drill-Tip Position Estimation During Cortical Bone Drilling Using Force and Torque Signals †

**DOI:** 10.3390/s26175319

**Published:** 2026-08-22

**Authors:** Hirotatsu Imai, Han Wang, Koki Kishimoto, Kosuke Kita, Yuki Suzuki, Koki Hosozawa, Yuya Kanie, Masayuki Furuya, Toshiyuki Enomoto, Seiji Okada, Takahito Fujimori

**Affiliations:** 1 Department of Orthopaedic Surgery, The University of Osaka Graduate School of Medicine, 2-2 Yamadaoka, Suita 565-0871, Japan; hirotatsu.imai.0722@gmail.com (H.I.);; 2 Department of Orthopaedic Surgery, Bellland General Hospital, Sakai 599-8247, Japan; 3 Department of Mechanical Engineering, The University of Osaka Graduate School of Engineering, Suita 565-0871, Japan; 4 Department of Artificial Intelligence Diagnostic Radiology, The University of Osaka Graduate School of Medicine, Suita 565-0871, Japan; 5 Health and Counseling Center, The University of Osaka, Toyonaka 560-0043, Japan

**Keywords:** cortical bone drilling, drill-tip position regression, force–torque sensing, deep learning, physical artificial intelligence

## Abstract

Purpose: Excessive drill advancement after cortical breakthrough is a potential safety concern in orthopaedic procedures. We developed a data-driven approach to estimate the drill-tip position relative to the far cortex prior to breakthrough using time-series thrust force and spindle torque signals. Methods: Drilling experiments were performed on 268 porcine cortical bone specimens at a constant feed rate of 0.5 mm/s. A long short-term memory network was trained to estimate the drill-tip position from filtered force and torque signals. The reference position was derived from breakthrough timing confirmed by high-speed imaging and the programmed feed rate. Performance was evaluated using mean absolute error within the −2 to +2 mm peri-breakthrough interval. Two post hoc analyses examined whether model performance exceeded an elapsed-time baseline and whether pre-breakthrough force patterns were more consistent when expressed relative to breakthrough position than to drilling onset time. Results: The combined-input LSTM achieved an MAE of 0.20 mm, compared with 0.23 mm for force alone and 0.24 mm for torque alone. Among the representative architectures evaluated, LSTM showed the lowest regression error. A signal-blind time-only baseline yielded an MAE of 0.54 mm. The association between cortical thickness and force-decline onset was weaker when expressed in spatial coordinates relative to breakthrough than when expressed as time from drilling onset (R^2^ = 23% vs. 74%). These findings suggest that force and torque signals contained information associated with proximity to breakthrough beyond that provided by average drilling duration alone. Conclusion: Converting sensor-derived resistance patterns into spatially anchored positional information may support proactive strategies such as controlled deceleration before penetration. The proposed approach represents a step toward exemplifying the emerging concept of surgeon-assisting Physical AI.

## 1. Introduction

Bone drilling is a fundamental technique in orthopaedic surgery, used in procedures such as fracture fixation, arthroplasty, and spinal instrumentation. Despite its ubiquity, a major technical risk is excessive drill advancement after cortical breakthrough. Even minimal overshoot can injure neurovascular or soft-tissue structures, especially near critical regions such as the spinal canal or joint surfaces [1,2]. Experienced surgeons rely on tactile feedback to anticipate cortical penetration, but this skill requires substantial experience. For less experienced operators, or when tactile feedback is reduced—such as during power-assisted or robotic drilling—the risk of iatrogenic injury may be increased [3].

To address this challenge, various mechanical and signal-based methods have been developed to improve the safety of cortical bone drilling [4,5,6]. Some neurosurgical perforators use clutch mechanisms that automatically disengage the drill upon penetration [7,8], while other systems monitor thrust force, torque, or acoustic emissions to detect the sudden drop in resistance associated with cortical breakthrough [4,5,6,9,10]. In parallel, vibrational and ultrasonic drilling techniques have been proposed as alternative strategies to reduce cutting forces and the risk of overshoot [11]. Most of these approaches, however, focus on detecting breakthrough as a discrete event and therefore provide limited information about the ongoing progression of the drill tip before penetration. Lang and Gilmer developed a dual-motor drill that simultaneously measures torque and drilling depth, enabling continuous monitoring of drill advancement and reducing overpenetration in a bone-block model [12]. This approach obtains depth information through direct mechanical measurement of drill advancement. In contrast, estimating the drill-tip position relative to the far cortex from force and torque signals alone, without direct depth measurement as an input, remains insufficiently explored. Such estimation could provide anticipatory information before breakthrough and potentially support controlled deceleration or stopping as the drill approaches the far cortex. In addition, signal-based approaches may be affected by variations in bone quality, density, geometry, and drilling conditions, making robustness across anatomical and procedural variability an important consideration [4].

In parallel with these mechanical and sensing-based advances, recent years have seen rapid growth in artificial intelligence (AI) applications in orthopaedics. Deep learning has been applied to image-based diagnosis, pathological assessment, automated radiographic measurements, and analysis of patient-reported questionnaires, demonstrating broad clinical and research utility [13,14,15]. Building on these achievements, interest has shifted toward intraoperative applications, where sensor data can enhance surgical safety and precision. Several studies have shown that force, torque, and acoustic signals collected during drilling contain rich information about underlying bone structures [9,10,16]. However, most reported approaches have focused on discrete classification of cortical versus cancellous bone or event-based detection of breakthrough, rather than continuous estimation of drill progression or proximity to cortical breakthrough.

From a surgical safety perspective, the ability to continuously estimate the remaining cortical thickness before breakthrough would represent a major advance. Real-time awareness of the drill-tip position relative to the far cortex could allow proactive adjustments—such as controlled deceleration, automatic pause, or haptic feedback—to prevent overshoot. Such technology would be particularly beneficial in minimally invasive or robotic-assisted orthopaedic procedures, where tactile feedback is diminished and automated safety mechanisms are critical.

In this context, we developed a deep learning model capable of continuously estimating drill-tip position relative to the far cortex from time-series force and torque signals. Although force and torque sensing is not standard in current orthopaedic hand drills, such sensing can be integrated into robotic or smart drilling platforms, providing a potential pathway for implementation of the proposed approach. By formulating the problem as a temporal regression task using a long short-term memory (LSTM) network, the model aims to emulate the anticipatory judgment of an experienced surgeon through data-driven learning of force–torque dynamics. The primary objective of this proof-of-concept study was to determine whether such a model could achieve sub-millimetre accuracy within the clinically critical peri-breakthrough zone, and whether its predictions reflect signal-derived spatial information rather than the elapsed drilling time.

## 2. Materials and Methods

### 2.1. Study Design and Ethical Considerations

This study was an in vitro experimental investigation designed to evaluate whether a deep learning model could continuously estimate drill-tip position from force and torque signals during cortical bone drilling. Porcine ribs were obtained from a commercial food supplier and used as non-living specimens for laboratory testing. As the study did not involve live animals or human participants, formal ethical approval was not required in accordance with institutional and national regulations.

### 2.2. Specimen Preparation

**Figure 1:**
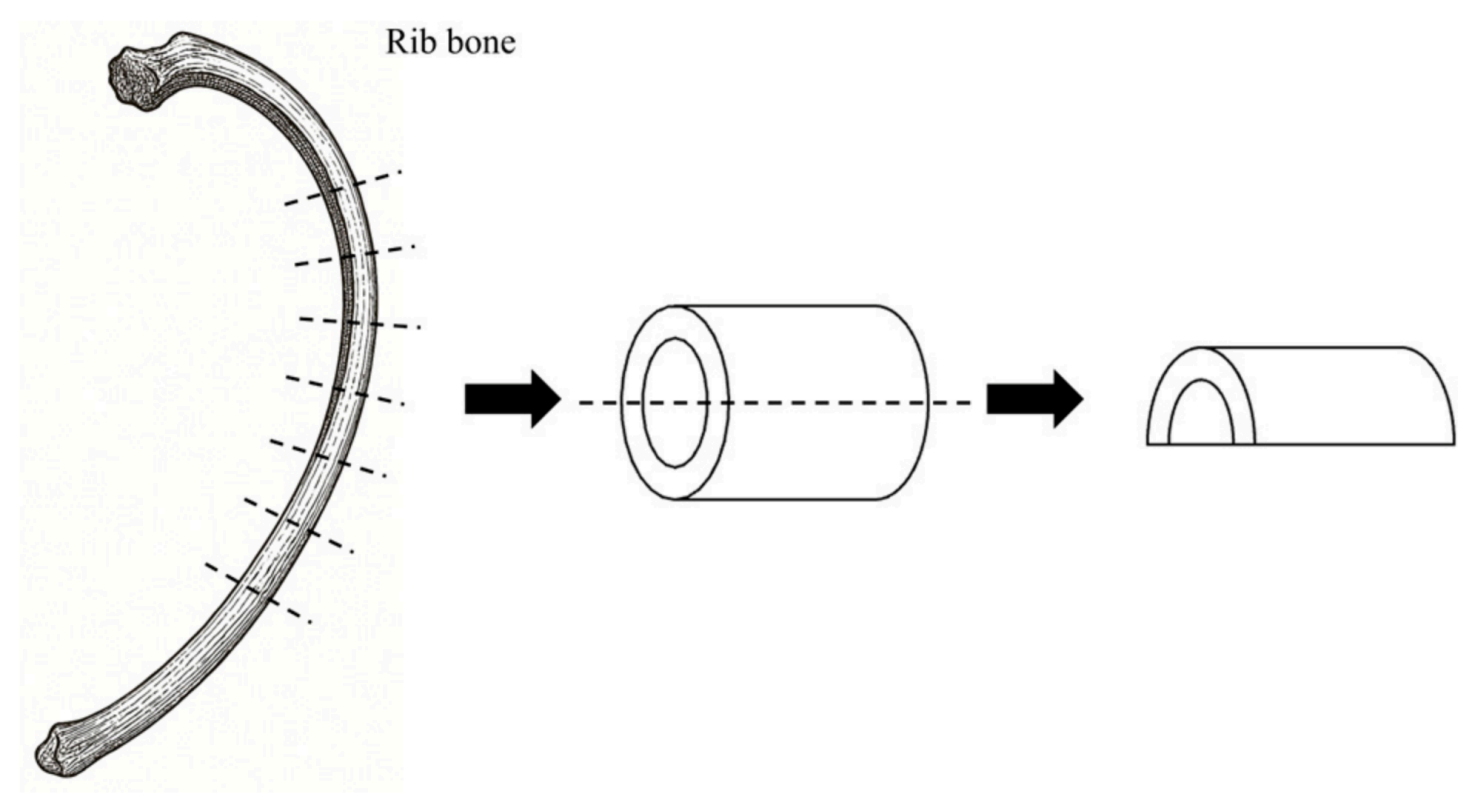
Preparation of porcine cortical rib specimens. Porcine rib shafts were sectioned at 2 cm intervals (short axis) and split longitudinally; cancellous bone was removed to yield cortical segments ≥ 2 mm in thickness (mean 3.0 ± 0.6 mm; range, 2.0–5.2 mm). These specimens were used for subsequent drilling experiments.

Approximately 20–30 commercially available dried porcine ribs intended for animal feed were used. Because the ribs were commercially sourced, information regarding the breed, age, sex, health status, and other donor-specific characteristics of the pigs was unavailable. Each rib was sectioned into 2 cm segments along the short axis and subsequently split longitudinally, yielding approximately 10 specimens per rib. The cancellous bone was removed, and cortical segments with a minimum thickness of 2 mm were selected (mean thickness, 3.0 ± 0.6 mm; range, 2.0–5.2 mm) (Figure 1). No additional inclusion or exclusion criteria were applied based on rib location, gross appearance, or presumed bone quality. No radiographic, densitometric, or histological screening was performed to confirm the absence of skeletal pathology or to characterize bone quality before the drilling experiments. In total, 268 cortical bone specimens were prepared. Each specimen was rigidly mounted in a custom-made jig to maintain a perpendicular drilling angle and minimize vibration during drilling.

### 2.3. Equipment

**Figure 2:**
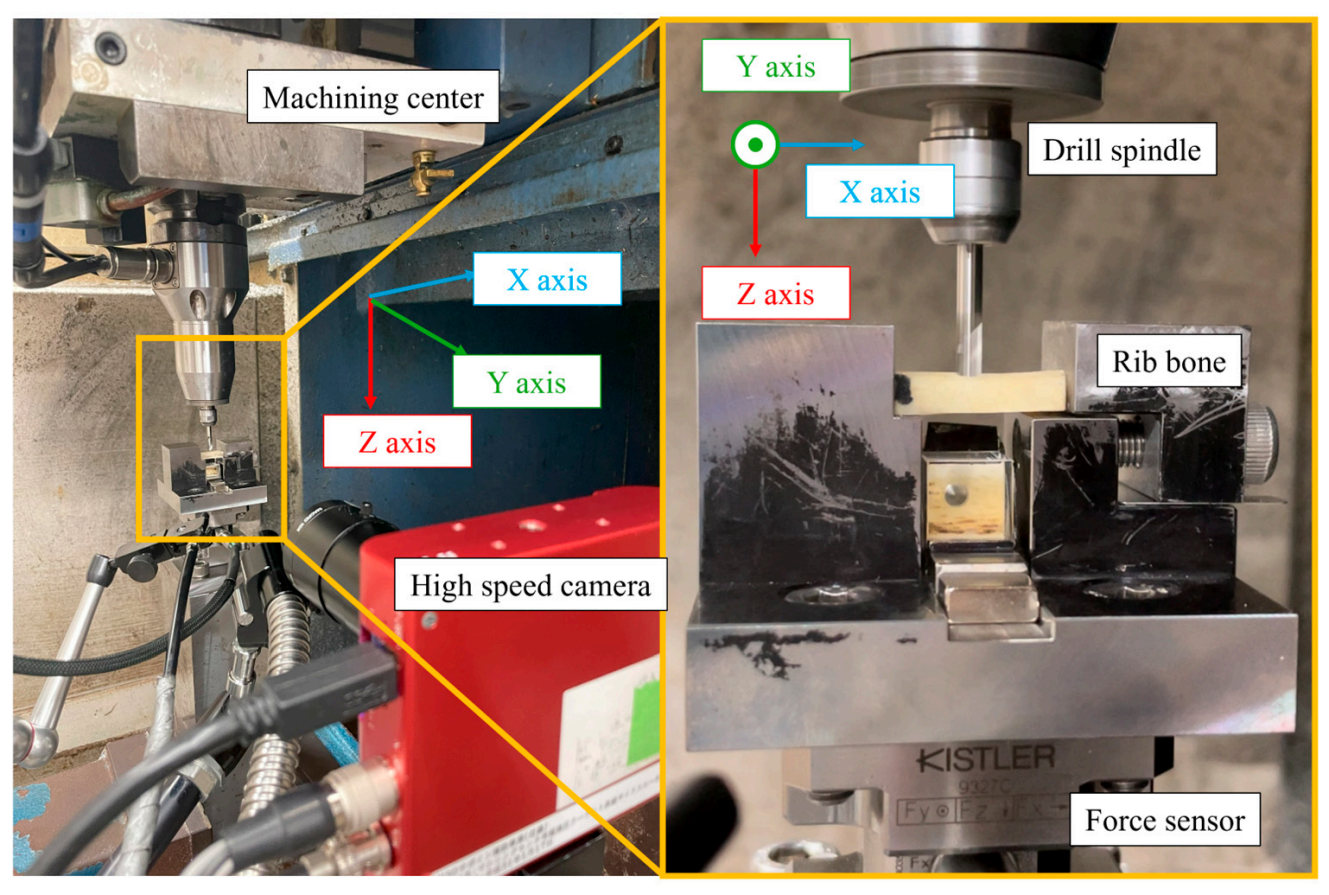
Experimental setup for cortical bone drilling. A drill spindle was mounted on a machining centre with a 3 mm twist drill bit. The specimen jig was fixed on a force sensor to record thrust force and torque. Cortical breakthrough was confirmed by observing the opposite surface with a high-speed camera via a mirror.

Drilling experiments were performed using a computer-controlled machining centre (AJV-18; Yamazaki Mazak Corp., Niwa, Japan) equipped with a high-speed spindle (HES810; NAKANISHI Inc., Kanuma, Japan) and a twist drill bit (MISUMI Corporation, Chiyoda, Japan). The drill bit had a diameter of 3.0 mm, a shank diameter of 3.0 mm, a point angle of 90°, and a flute length of 10 mm. The overall experimental setup is shown in Figure 2.

Each cortical specimen was mounted in a custom-designed jig and rigidly fixed onto a six-axis force sensor (9327C; Kistler Group, Winterthur, Switzerland), which recorded thrust force along the Z-axis. The jig–sensor assembly was positioned beneath the drill spindle to ensure perpendicular drilling under controlled feed conditions. Spindle torque was derived from the motor load voltage signal of the drilling unit.

Cortical breakthrough was visually confirmed using a high-speed camera (HAS-EF; DITECT Co., Ltd., Shibuya, Japan) operating at 100 frames per second. The camera was positioned on the side opposite the drill exit surface, and a mirror was used to obtain a clear view of the far cortex. The high-speed imaging served as the ground-truth reference for breakthrough timing and drill-tip position annotation.

All data streams—including thrust force, spindle torque, and synchronization trigger signals—were simultaneously acquired using a data logger (NR-600; Keyence Corp., Higashiyodogawa, Japan) at a sampling rate of 10,000 Hz. Before each experimental session, the thrust-force and motor-load voltage signals were zeroed under no-load conditions. Spindle torque was estimated from the motor-load voltage using a predetermined voltage-to-torque conversion coefficient. All devices were synchronized using a shared trigger pulse to ensure precise temporal alignment between sensor data and high-speed imaging.

### 2.4. Experimental Protocol

**Figure 3:**
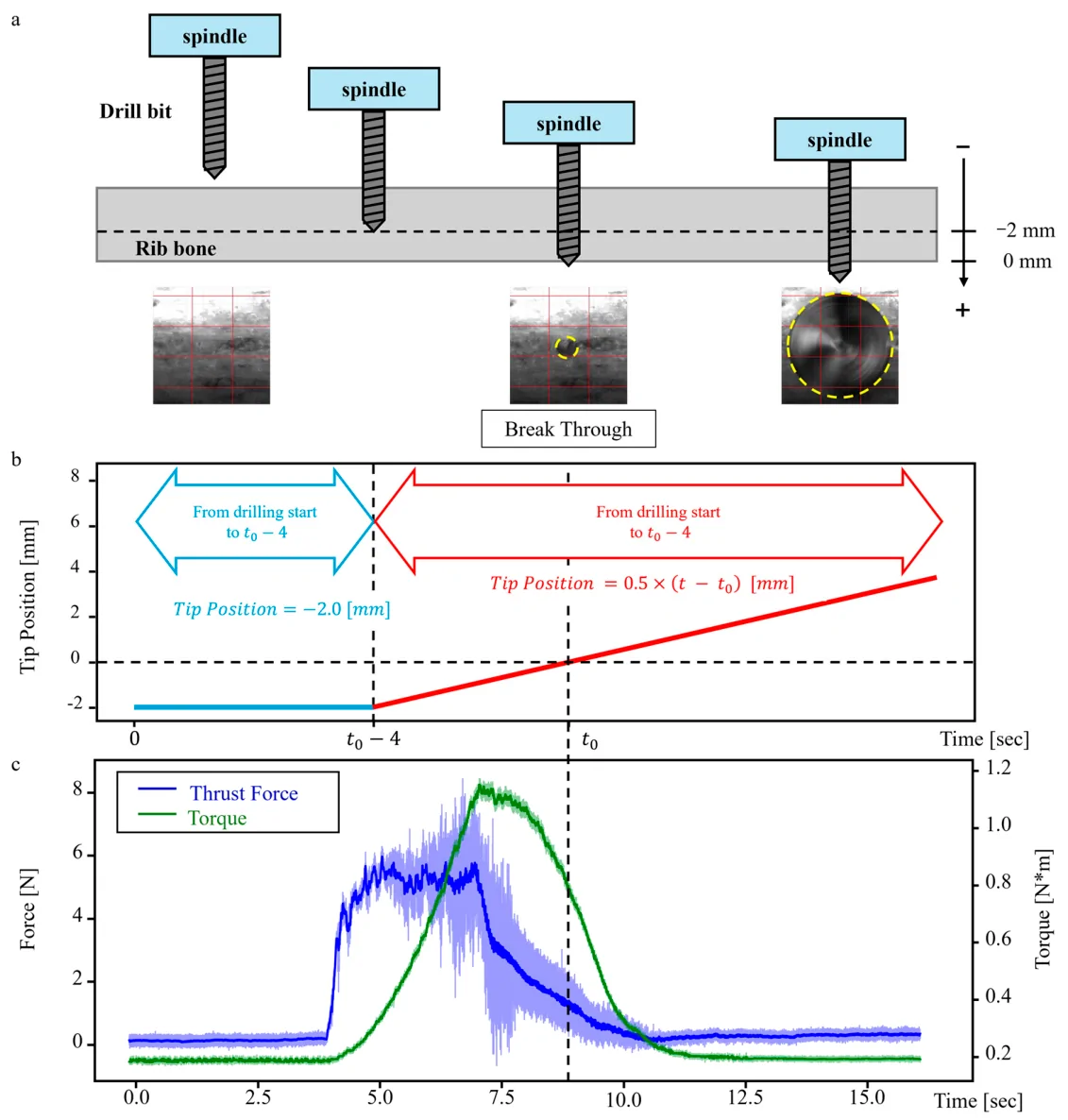
(**a**) Schematic of drill progression in a porcine rib; the direction of advancement (downward) is defined as the positive Z-axis. High-speed camera views of the opposite cortex: pre-breakthrough (tip not visible), breakthrough at $t_{0}$(first appearance of the tip), and complete breakthrough (full drill diameter visible). The yellow dashed circles indicate the visible drill tip at breakthrough and the full drill diameter after complete breakthrough. (**b**) Ground-truth labelling for deep learning. Tip position is defined piecewise with −2 mm for $t\leq t_{-2}\;(t_{-2}=\;t_{0}-4\;s)$ and $0.5\;\times \left(t-\;t_{0}\right)\;\mathrm{mm}$ for $t\;\geq \;t_{-2}$. (**c**) Representative raw and filtered time-series signals during drilling: thrust force (blue) and torque (green).

Each of the 268 cortical bone specimens was drilled once, resulting in a total of 268 drilling trials (268 holes). The drill was positioned approximately 5 mm above the cortical surface and advanced perpendicularly under uniform operating conditions for all trials at a constant spindle speed of 2000 rpm and a feed rate of 0.5 mm/s. Drilling was continued until the far cortex was fully penetrated, corresponding to a total advancement of 15 mm, ensuring complete penetration through the cortical segment. Breakthrough was defined as the moment the drill tip first appeared beyond the far cortex on high-speed imaging. All force, torque, and imaging signals were recorded concurrently throughout each trial. Drill progression and breakthrough detection are illustrated in Figure 3a.

### 2.5. Data Processing

Raw thrust force F(t) and torque $T\left(t\right)$ signals were processed before model training. A fourth-order Butterworth low-pass filter with a cut-off frequency of 50 Hz was applied to remove high-frequency noise. Signals were then standardized on a per-trial basis using Equation (1).

(1) $$x_{std}\left(t\right)=\frac{x(t)-\mu }{\sigma }\;$$

where x(t) represents either the filtered thrust-force or torque signal, and μ and σ are the mean and standard deviation, respectively, of the corresponding signal calculated over the entire recorded sequence of each trial. Thus, thrust force and torque were standardized separately for each trial. To align with the temporal resolution of the high-speed camera and reduce computational load, the filtered signals were downsampled from 10,000 Hz to 100 Hz by keeping one sample out of every 100 consecutive samples and discarding the remaining 99 samples. No averaging or interpolation was performed.

### 2.6. Drill-Tip Position Annotation for Regression

The time of breakthrough, defined on camera as the first appearance of the drill tip beyond the far cortex, was denoted $t_{0}$. Drill-tip position was annotated relative to this event using a piecewise definition (Equation (2)):

(2) $$Drill-tip\;Position\;(t)=\;\left\{\begin{array}{c} -2.0\;\left(t\;\leq \;t_{-2}\right)\\ 0.5\;\times \left(t-\;t_{0}\right)\;\left(t>t_{-2}\right) \end{array}\right.$$

where $t_{-2}=\;t_{0}-4\;s$ corresponds to 2 mm before breakthrough, given the constant feed rate of 0.5 mm/s. Accordingly, we defined the tip position as −2.0 mm before this point, and as increasing linearly after this point, with 0 mm at $t_{0}$. Position values continued positively thereafter. This annotation process is illustrated in Figure 3b. Model evaluation was restricted to the peri-breakthrough interval of −2 to +2 mm, as this region is clinically critical for preventing overshoot and ensuring precise control of drilling [17,18,19]. No separate quantitative evaluation was performed outside this interval. Positions earlier than −2 mm were assigned a constant target value of −2 mm, and the amount of available data outside this boundary varied with cortical thickness. Therefore, extending the evaluation beyond this standardized interval could bias performance estimates and disproportionately weight thicker specimens. Model performance outside the ±2 mm interval remains unknown.

### 2.7. Penetration Annotation for Binary Classification

Each time point within the ±2 mm peri-breakthrough interval was labeled as either pre-penetration or post-penetration based on the breakthrough timing ($t_{0}$) defined in the Experimental protocol. These labels were used to train the LSTM classifier.

### 2.8. Outcome Measures

Several drilling parameters were defined from synchronized signals and camera:

- Drill-tip position (mm): As defined in Equation (2).
- Baseline force (N): The mean thrust force during the 1 s interval before drilling onset.
- Drilling start time (sec): The point when the thrust force exceeded three standard deviations above baseline, corresponding to initial bone contact.
- Breakthrough time (sec): The reference time $t_{0}$.
- Drilling duration (sec): The interval between drilling start and breakthrough.
- Maximum thrust force (N): The peak thrust force recorded during drilling.
- Maximum torque (N·m): The peak torque recorded during drilling.

These definitions were applied consistently across all trials and used in both descriptive analysis and model evaluation.

### 2.9. AI Model

**Figure 4:**
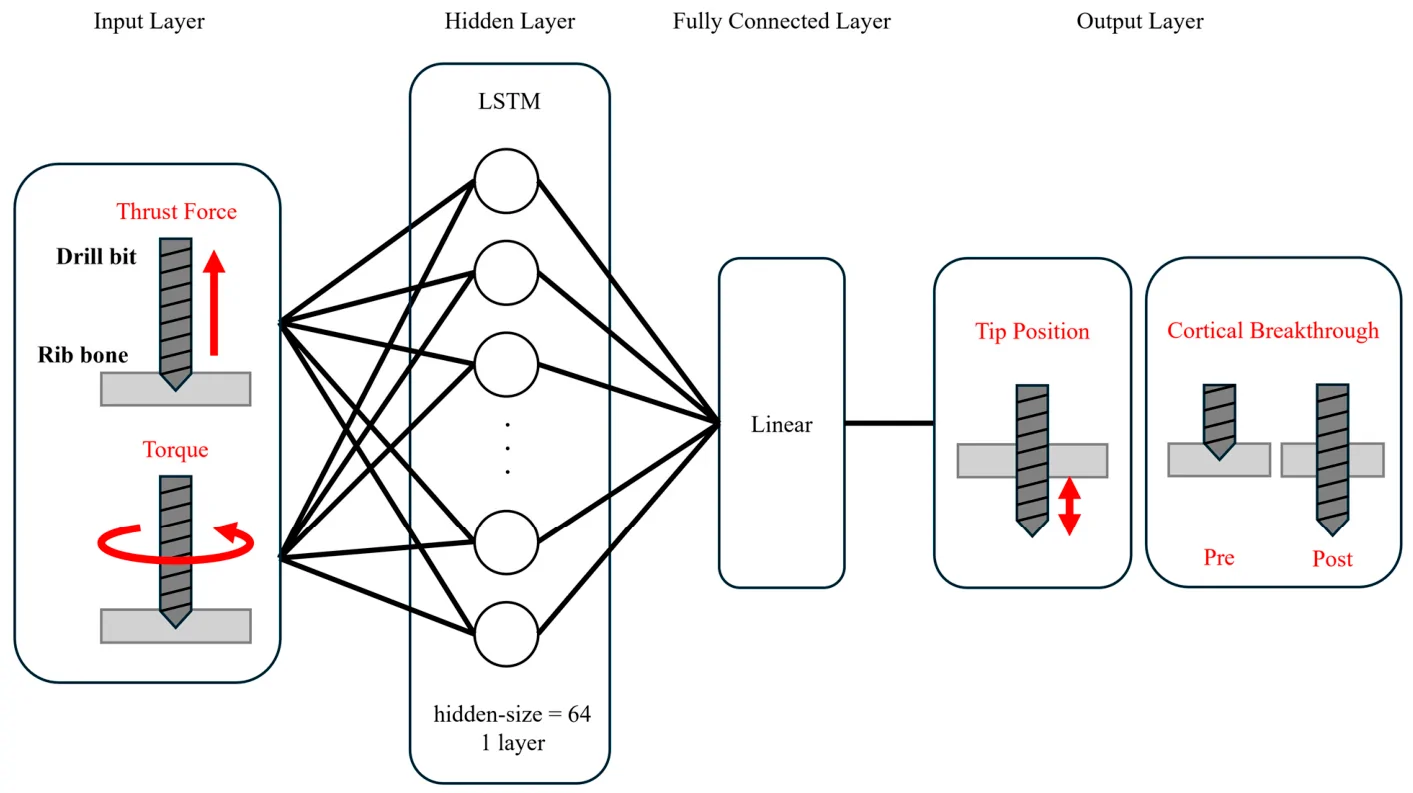
Architecture of the deep learning model. The input layer receives thrust force, torque, or both signals, which are passed through a single LSTM layer with 64 units, followed by a fully connected layer that outputs the predicted drill-tip position.

An LSTM architecture was selected because drill-tip position was expected to depend on the temporal evolution of sequential thrust-force and torque signals rather than on isolated measurements. Its gated memory structure enables the model to capture nonlinear temporal dependencies while mitigating the vanishing-gradient problem, making it suitable for learning patterns such as the progressive rise, plateau, and decline of force and torque during cortical drilling. Two long short-term memory (LSTM) neural network models were developed: one for continuous regression of drill-tip position and one for binary classification of cortical breakthrough. Both models used the same preprocessing pipeline, input configurations (force only, torque only, or combined force–torque), and overall network architecture to enable consistent comparison between continuous estimation and discrete detection. A schematic overview of the model architecture is shown in Figure 4.

The network consisted of a single-layer LSTM with 64 hidden units followed by a fully connected output layer. The dataset of 268 trials was divided by specimen into training (60%), validation (20%), and test (20%) sets to prevent data leakage.

To assess whether model performance was specific to the LSTM architecture, three comparator models were also evaluated: a linear model, a temporal convolutional network (TCN), and a gated recurrent unit (GRU) network. For architecture benchmarking, all models used the combined force–torque input and were evaluated using the same preprocessed data, data partition, and ±2 mm peri-breakthrough interval. Regression performance was compared using MAE and RMSE, whereas classification performance was compared using accuracy, precision, recall, F1-score, and AUC.

For the regression task, the network outputs a continuous estimate of drill-tip position and was trained using mean squared error loss. Model performance was evaluated using mean absolute error (MAE) within the ±2 mm peri-breakthrough interval. MAE was selected as the primary metric because it provides an intuitive measure of the average drill-tip position error directly in millimeters. Because the clinical objective may be either to stop immediately after far-cortex penetration or to advance the drill as close as possible to the far cortex without penetration, both overestimation and underestimation are clinically relevant. Accordingly, MAE was used as a balanced measure of overall positional accuracy.

For the classification task, time points within the same ±2 mm peri-breakthrough interval were extracted and labeled based on the breakthrough timing defined in the Experimental protocol. Samples acquired before breakthrough were labeled as 0 (pre-penetration), and those acquired after breakthrough were labeled as 1 (post-penetration). The model was trained using binary cross-entropy loss, and classification performance was assessed using accuracy, precision, recall, F1-score, and AUC within this interval.

Models were trained for 300 epochs using the Adam optimizer with an initial learning rate of 0.01, which was adaptively reduced when validation loss plateaued. All models were implemented in PyTorch (v2.3.1) using Python (v3.8.19) and trained on an NVIDIA RTX 4060 GPU. The source code for model training and evaluation is publicly available at: https://github.com/hirotatsuimai0722-coder/models (accessed on 10 August 2026).

### 2.10. Statistical Analysis

The primary evaluation metrics for the regression task were MAE between predicted and ground-truth positions, calculated within the peri-breakthrough interval of [−2, +2] mm. For the binary classification task, model performance was evaluated within the same ±2 mm peri-breakthrough interval using accuracy, precision, recall, F1-score, and AUC. Descriptive statistics of drilling parameters (drilling duration, baseline force, maximum thrust force, maximum torque) were reported as mean values with standard deviations, together with minimum, maximum, and interquartile ranges. A significance level of 5% was used. No missing data was encountered.

### 2.11. Analyses to Dissociate Signal-Based Estimation from Elapsed-Time Learning

Because all trials were performed at a constant feed rate, drill-tip position and elapsed time relative to breakthrough are inherently collinear. To assess whether the LSTM extracted signal-derived spatial information rather than a stereotyped temporal pattern, two complementary post hoc analyses were performed on the full dataset (*n* = 268 trials).

### 2.12. Time-Only Baseline Analysis

A signal-blind baseline predicted breakthrough timing using only the drilling onset time and the leave-one-out mean drilling duration. Position predictions were generated using the same definition as the ground truth, substituting the estimated breakthrough time for the observed breakthrough time. MAE was calculated within the ±2 mm peri-breakthrough interval and compared with that of the combined-input LSTM on the same set of trials.

### 2.13. Relationship of Pre-Breakthrough Force Decline with Cortical Thickness and Drilling Time

Filtered thrust force traces (Fz; 4th-order Butterworth, 50 Hz cut-off) were aligned in spatial coordinates by mapping time to position relative to breakthrough as $p\left(t\right)=0.5\times (t\;-\;t_{0})$ mm, sampled on a 0.01 mm grid from −2.5 to +0.5 mm. Trials were stratified into tertiles by cortical thickness (estimated as 0.5 × drilling duration). At each spatial bin of 0.25 mm, inter-group differences were tested with the Kruskal–Wallis test. For each trial, a decline-onset position was defined as the position at which the filtered thrust force, decreasing from its plateau (mean Fz over [−2.5, −1.5] mm), first crossed the half-amplitude threshold defined relative to the value at breakthrough. The decline-onset position was regressed against cortical thickness, and the same event expressed in time-from-onset coordinates was regressed against thickness for comparison. Under the 0.5 mm/s feed rate, a spatial slope of 0 mm/mm and a temporal slope of +2 s/mm both correspond to pure spatial anchoring, whereas slopes of −1 mm/mm and 0 s/mm correspond to pure depth-from-onset anchoring.

## 3. Results

### 3.1. Time-Series Changes in Force and Torque

A representative example of raw and filtered thrust force and torque signals is shown in Figure 3c. As drilling progressed, thrust force gradually increased and reached its peak just before breakthrough. Torque demonstrated smaller fluctuations compared with force but similarly showed a peak preceding breakthrough. After breakthrough, both thrust force and torque dropped sharply. These characteristic time-series patterns were consistently observed across specimens.

### 3.2. Drilling Parameters

The statistical distribution of the key parameters is presented in Table 1. The median drilling duration was 5.80 s (IQR 4.95–6.96; range 4.02–10.45). The median maximum thrust force was 7.29 N (IQR 5.96–8.36; range 1.81–12.79), and the median maximum torque was 1.32 N·m (IQR 1.09–1.61; range 0.43–3.01).

### 3.3. AI Model Performance: Regression

**Figure 5:**
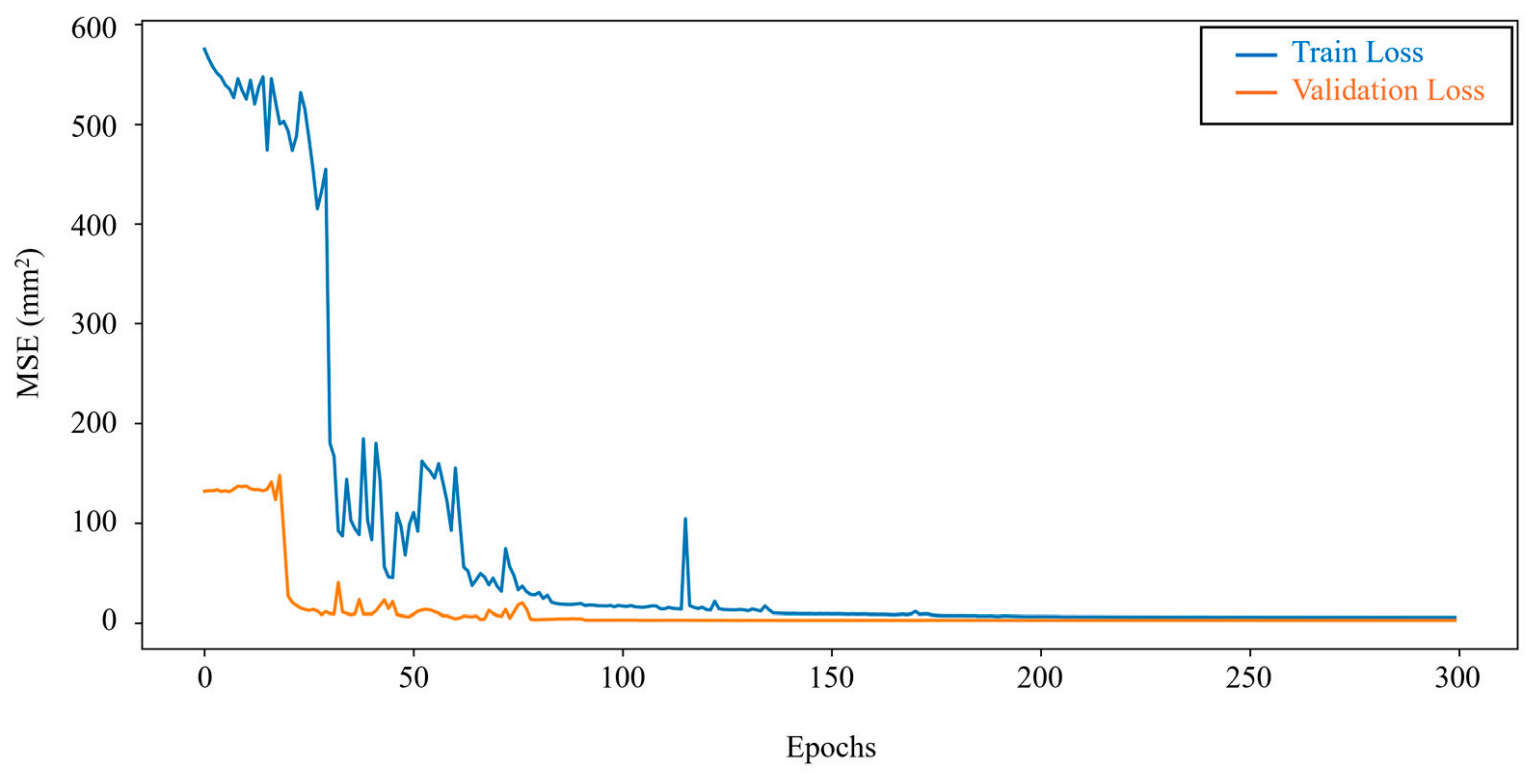
Learning curve of the LSTM regression model using both thrust force and torque as inputs. Training and validation losses (mean squared error) decreased steadily over the initial epochs and converged at approximately 100 epochs, indicating stable optimization without evidence of overfitting.

**Figure 6:**
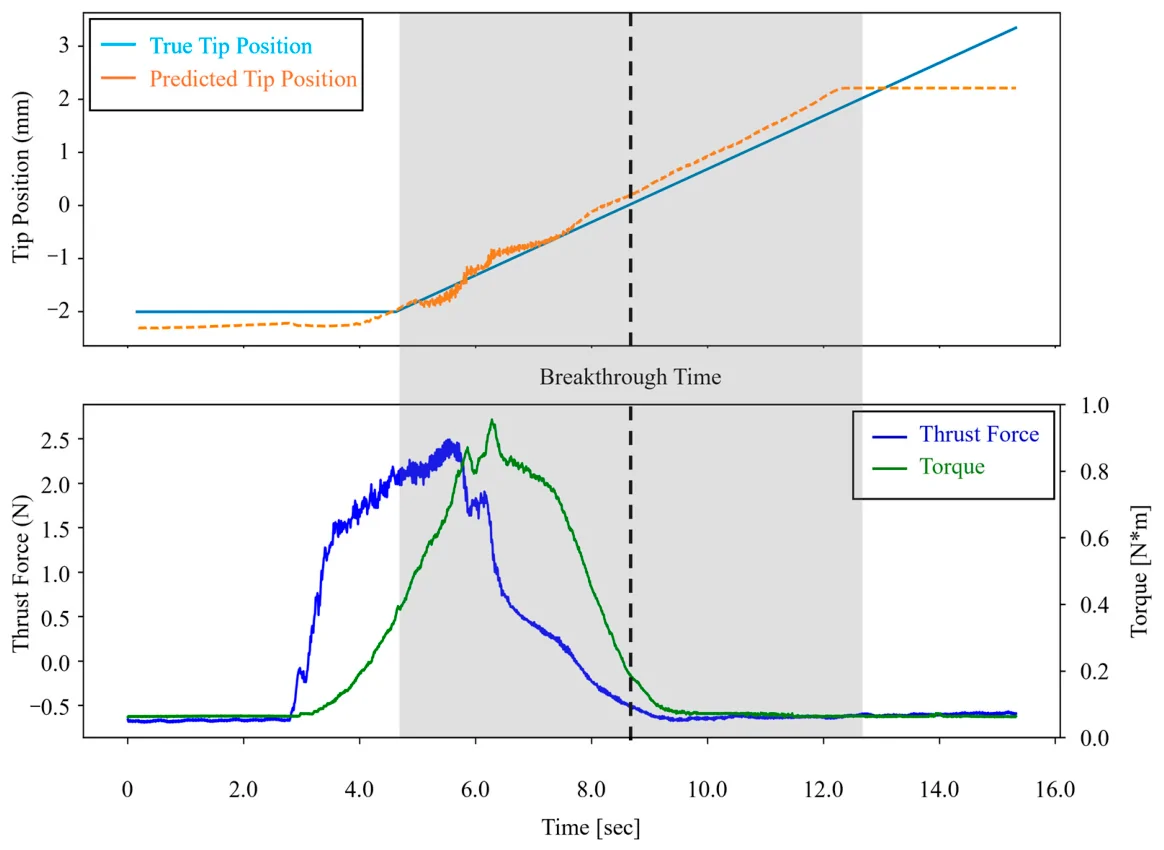
Representative test case showing predicted (orange line) and ground-truth drill-tip positions within the ±2 mm peri-breakthrough interval. The predicted trajectory closely follows the ground-truth position, demonstrating accurate tracking of drill advancement relative to the far cortex near breakthrough. The vertical dashed line indicates the breakthrough time, and the shaded region denotes the ±2 mm peri-breakthrough evaluation interval.

Using the LSTM model, the combined force–torque input achieved an MAE of 0.197 mm and an RMSE of 0.228 mm. The MAE was 0.230 mm for thrust force alone and 0.245 mm for torque alone. In the architecture comparison using the combined force–torque input, LSTM achieved the lowest regression error, followed by GRU, TCN, and the linear model (Table 2). Training convergence of the LSTM model is shown in Figure 5, and a representative test case is shown in Figure 6.

### 3.4. AI Model Performance: Binary Classification

Using the combined force–torque input, the LSTM classifier achieved an accuracy of 0.934, precision of 0.943, recall of 0.935, F1-score of 0.931, and an AUC of 0.999. Performance according to LSTM input configuration is summarized in Table 3. In the architecture comparison using the combined force–torque input (Table 2), GRU achieved the highest accuracy (0.944) and F1-score (0.943), whereas LSTM achieved the highest AUC (0.999). TCN and the linear model showed lower overall classification performance. These findings indicate that the relative performance of the evaluated architectures differed between the regression and classification tasks.

**Figure 7:**
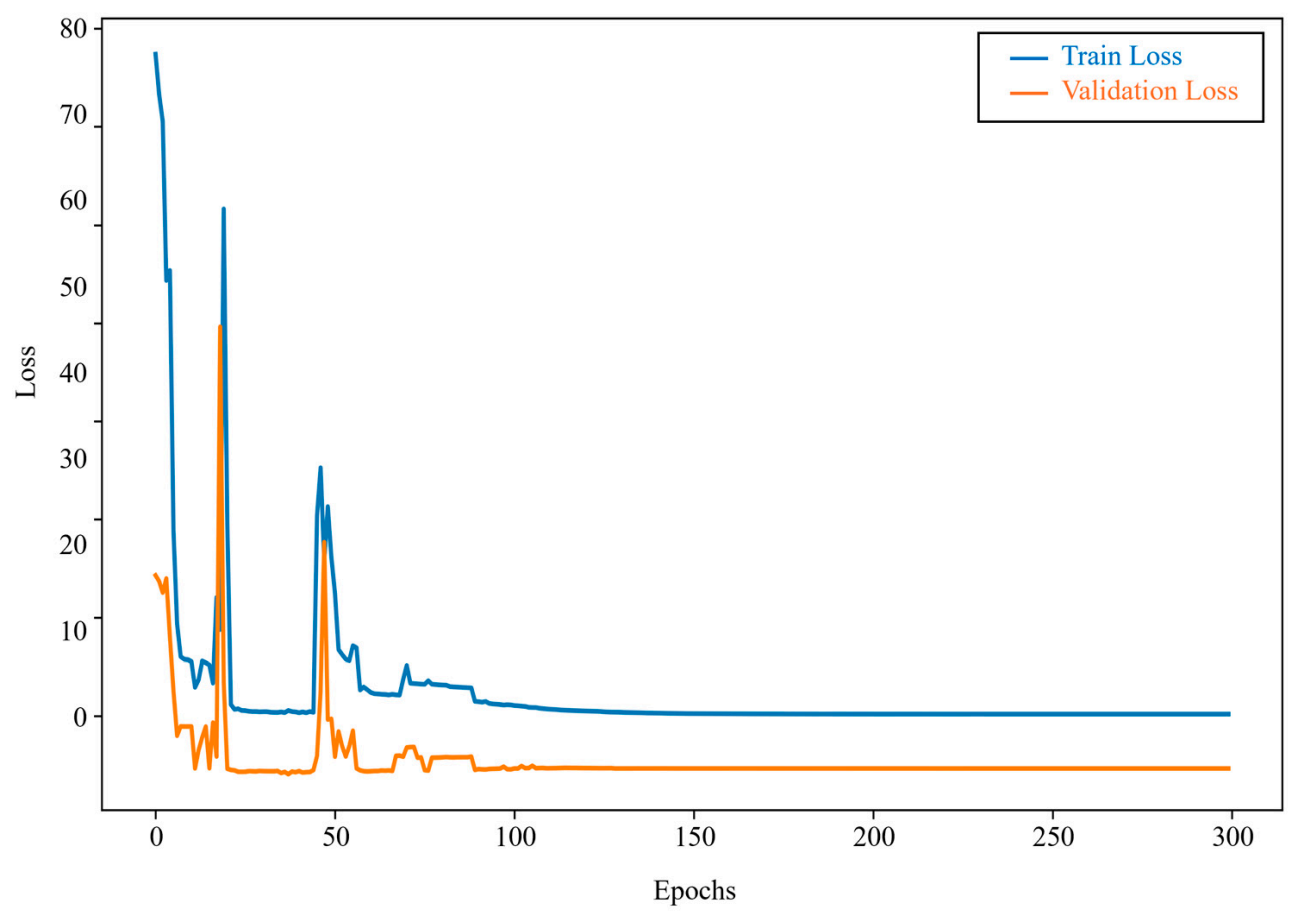
Learning curve of the LSTM binary classification model using combined thrust force and torque inputs. Training and validation losses (binary cross-entropy) decreased rapidly during the early epochs and plateaued around 60 epochs, indicating stable convergence without overfitting.

**Figure 8:**
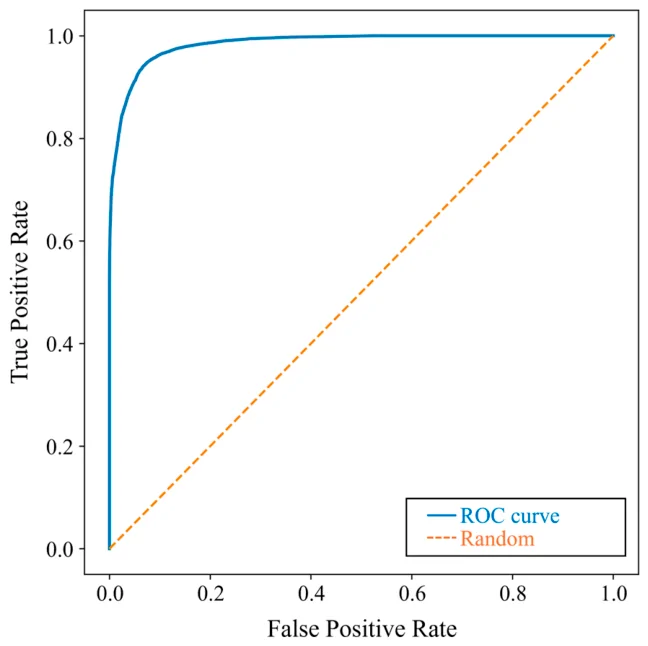
Receiver operating characteristic (ROC) curve of the combined-input LSTM classifier within the ±2 mm peri-breakthrough interval. The curve approaches the upper-left corner, with an area under the curve (AUC) of 0.999, demonstrating excellent discriminative performance for identifying cortical breakthrough from force–torque signals.

The learning curve of the dual-variable classification model showed a rapid decline in training and validation losses during the early epochs, followed by stabilization around 60 epochs, reflecting a convergence pattern similar to that observed in the regression task (Figure 7). The receiver operating characteristic (ROC) curve illustrates the trade-off between sensitivity and the false-positive rate across classification thresholds, whereas the area under the curve (AUC) summarizes overall discriminative performance. The ROC curve for the combined-input model demonstrated excellent discriminative ability, with an AUC of 0.999 (Figure 8).

### 3.5. Dissociation of Signal-Based Estimation from Elapsed-Time Learning

#### 3.5.1. Performance of the Time-Only Baseline

The signal-blind time-only baseline achieved an MAE of 0.54 mm within the ±2 mm peri-breakthrough interval, compared with 0.20 mm for the combined-input LSTM. The baseline error was strongly associated with the absolute deviation of each trial’s drilling duration from the mean duration (r=0.996).

#### 3.5.2. Spatial Anchoring of the Pre-Breakthrough Force Decline

**Figure 9:**
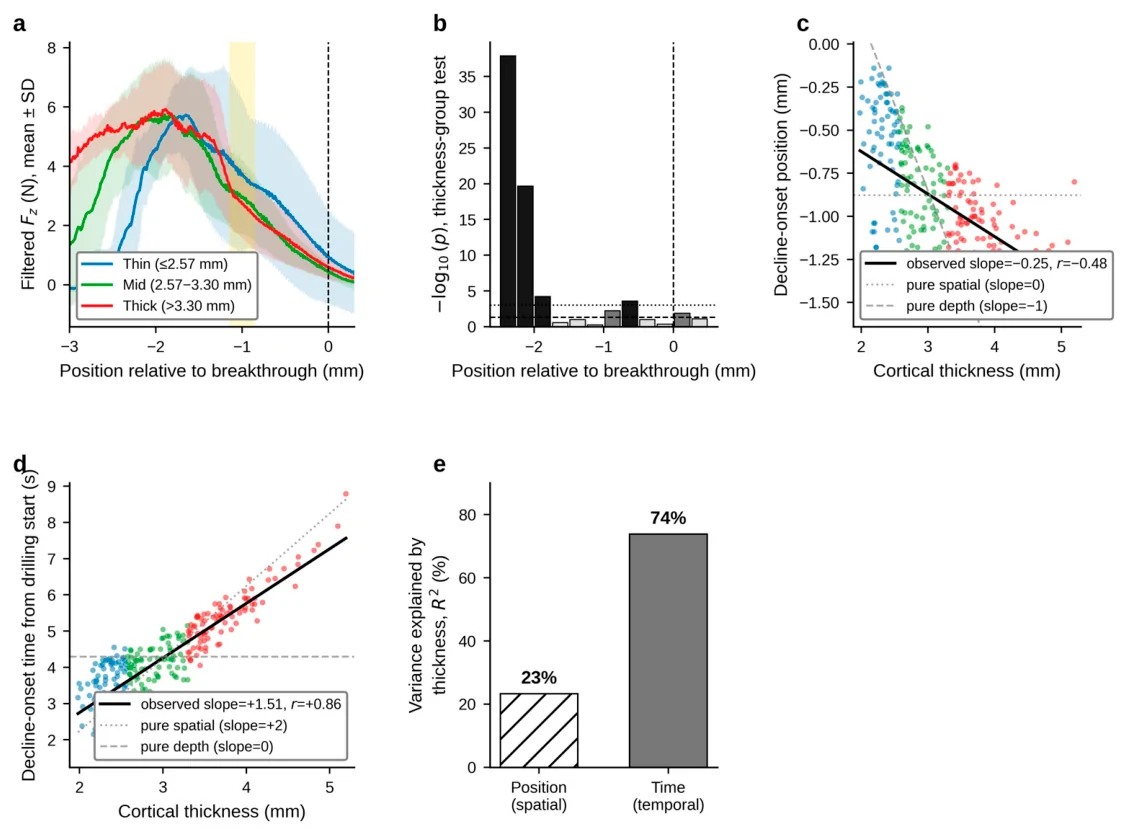
Pre-breakthrough force decline is anchored to remaining cortical thickness rather than to elapsed drilling time. (**a**) Mean ± SD filtered Fz by cortical-thickness tertile (thin/mid/thick), plotted against position relative to breakthrough; the gold band marks the convergence zone around −1 mm. Blue, green, and red indicate the thin, mid, and thick tertiles, respectively. (**b**) Per-bin Kruskal–Wallis tests across thickness groups; differences collapse to non-significance within approximately 1 mm of breakthrough. The vertical dashed line in (**a**,**b**) indicates the breakthrough position (0 mm). (**c**) Per-trial decline-onset position vs. cortical thickness, with reference lines for pure spatial anchoring (slope = 0) and pure depth-from-onset anchoring (slope = −1). (**d**) Same event expressed as decline-onset time from drilling start, showing strong thickness dependence. Colored dots in (**c**,**d**) represent individual trials according to the same thickness tertiles, and the dotted/dashed lines indicate the theoretical reference slopes shown in the legends. (**e**) Variance explained by thickness (R^2^) is approximately three-fold smaller in the spatial frame than in the temporal frame.

When filtered thrust force (Fz) traces were aligned to t_0_ and expressed in spatial coordinates, force profiles from thin, mid, and thick cortical specimens diverged far from breakthrough (Kruskal–Wallis *p* = 1.4 × 10^−39^ at −2.4 mm) but converged within approximately 1 mm of breakthrough (*p* = 0.37 at −1.1 mm and *p* = 0.44 at −0.9 mm; Figure 9). The decline-onset position depended only weakly on cortical thickness (Pearson r = −0.48, slope = −0.25 mm/mm, R^2^ = 23%), whereas the same event expressed in time-from-onset coordinates was strongly thickness-dependent (r = +0.86, slope = +1.51 s/mm, R^2^ = 74%). Switching from the temporal to the spatial frame reduced the thickness-explained variance approximately three-fold.

## 4. Discussion

This study presents a deep learning framework for continuous state estimation during cortical bone drilling, in which a long short-term memory (LSTM) network learned to infer drill-tip position directly from force–torque dynamics. Unlike previous threshold-based or classification approaches that detect cortical breakthrough only after it occurs, our model formulates the task as a temporal regression problem, enabling continuous estimation of drill-tip position under controlled experimental conditions. The proposed method achieved sub-millimetre accuracy (MAE 0.20 mm) within the ±2 mm peri-breakthrough zone, demonstrating that physical interaction signals alone can provide sufficiently rich information for anticipatory control.

### 4.1. Methodological and Conceptual Contributions

From a methodological perspective, this study extends conventional sensing-based drilling research from discrete event detection to continuous temporal estimation. By leveraging the LSTM architecture, the model captures the evolving temporal relationships between thrust force and torque as drilling progresses. This formulation differs from previous classification-based approaches by providing a continuous estimate of drill-tip position relative to the far cortex. Such positional information could potentially be incorporated into real-time feedback or robotic control systems.

### 4.2. Relation to Previous Research

Previous studies have primarily addressed cortical breakthrough as a discrete event, using force, torque, or acoustic signals as indicators. Early approaches relied on force- or energy-based thresholds to detect the sudden drop in resistance at penetration [4,5,6,20,21]. Subsequent efforts incorporated machine learning classifiers, such as those by Torun et al., which combined motor current and force signals to categorize drilling states with high accuracy [9]. More recently, Seibold et al. applied deep neural networks to acoustic data to identify penetration events in cadaveric specimens [10]. These studies demonstrated that sensor-derived signals contain sufficient information for reliable classification of cortical transitions. In the spinal surgery domain, our group similarly demonstrated that a machine learning model could detect bone penetration from intraoperative chisel percussion sounds, achieving robust discrimination on an independent test set [16]. Like other acoustic and force–torque approaches, however, this method classified penetration as a discrete event rather than continuously estimating drill-tip position. In our dataset, an LSTM-based classifier achieved similarly high performance, confirming that conventional classification of drilling states remains readily achievable using modern deep learning techniques. An important exception is the dual-motor drill reported by Lang and Gilmer, which simultaneously measures torque and drill advancement and thereby provides continuous depth information during drilling [12]. Unlike the present approach, however, positional information in their system is obtained through direct mechanical measurement of drill advancement rather than inferred from force–torque dynamics relative to the far cortex. A representative comparison of these sensing-based approaches and the continuous regression framework used in the present study is summarized in Table 4.

Importantly, the novelty of the present study does not lie in the use of force or torque sensing itself, as these signals have previously been used for robotic drilling, feedback control, and breakthrough detection [4,5,6,9,10,12]. Rather, the distinction lies in the formulation of the task: most signal-based approaches primarily identify a drilling state or breakthrough event, whereas the present model continuously estimates drill-tip position relative to the far cortex without direct depth measurement as an input. This continuously updated spatial estimate may provide information before penetration that is not available from a discrete breakthrough decision alone. Thus, although threshold-based and learned detection methods can reliably identify cortical transitions, they do not directly provide a continuous quantitative estimate of the remaining distance to the far cortex.

The present study therefore introduces a continuous regression framework that estimates drill-tip position from the temporal evolution of force–torque dynamics. This continuous estimation capability may support anticipatory rather than purely event-triggered feedback, opening opportunities for controlled deceleration or automated pause before excessive penetration. Because force and torque sensing can be incorporated into robotic or smart surgical platforms, the proposed approach may have translational potential, subject to further validation under clinically variable conditions.

### 4.3. Interpretation of the Learned Physical Dynamics

The predictive performance of the proposed model can be explained by the characteristic dynamics of thrust force and spindle torque, anchored in space relative to the far cortex. Both signals showed a gradual decrease immediately before cortical breakthrough, consistent with a reduction in apparent stiffness as the remaining cortical layer thinned. This reduction reflects elastic micro-deformation and a progressive loss of structural rigidity near the exit surface.

Because the feed rate was constant, drill-tip position and elapsed time move together, so one might suspect the LSTM simply learned how long drilling usually lasts before breakthrough—in effect, an internal stopwatch—rather than reading the signals. Two findings argue against this.

The first finding comes from comparing the LSTM with a deliberately signal-blind time-only model. Because the specimens varied in cortical thickness, drilling duration also varied across trials. The time-only model, which relied on the mean drilling duration, yielded a higher MAE than the combined-input LSTM (0.54 vs. 0.20 mm), and its error was strongly associated with each trial’s deviation from the mean drilling duration (r=0.996). These findings indicate that average elapsed time alone could not account for the model’s performance and support the interpretation that the LSTM extracted additional positional information from the force and torque signals.

The second finding identifies what that information may represent. As the far cortex thins, it begins to flex and the thrust force starts to fall. We found that this force decline was more consistently aligned with distance to breakthrough than with elapsed time under the present experimental conditions: when traces from thin, mid, and thick specimens were lined up by position, they converged within about 1 mm of breakthrough regardless of thickness, and the onset of the decline depended only weakly on thickness in spatial terms (R^2^ = 23%) but strongly in temporal terms (R^2^ = 74%). In other words, the force decline occurred at a relatively consistent distance from the far cortex rather than at a fixed time after drilling onset. This pattern provides a spatially informative signal feature, potentially reflecting micro-deformation of the thinning distal cortex, that may contribute to estimation of the remaining distance. The spatial anchoring was strong but not absolute (a residual spatial slope of about −0.25 mm/mm), so a small part of the signal still followed depth from onset; even so, taken together, the two analyses support the interpretation that the LSTM used space-dependent changes in the force–torque signals in addition to temporal information, although these contributions could not be fully separated under the constant-feed design.

### 4.4. Generalization and Potential Applications

Beyond the immediate task of bone drilling, the proposed framework illustrates a broader paradigm of “Physical AI”—artificial intelligence that learns from physical interactions captured by sensors. The same principle could be extended to other surgical actions, including cutting and milling, where force or vibration signals encode material transitions. Integration of this continuous estimation framework with real-time feedback control systems could enable autonomous or semi-autonomous surgical robots to anticipate critical transitions and adapt their behavior proactively. However, the practical advantage of this approach remains to be established. The present study did not directly compare the proposed model with conventional breakthrough-detection methods or motor-load-based feedback control, nor did it evaluate inference latency or closed-loop stopping performance. Future studies should compare these approaches under identical drilling conditions and assess whether continuous model-based position estimation, either alone or in combination with conventional feedback control, can reduce stopping time or overshoot. Moreover, incorporating additional sensor modalities such as vibration or acoustic emissions could further improve robustness and generalizability across different surgical contexts and anatomical sites. Clinical implementation would require calibrated and synchronized force–torque sensing within the drilling system. Such integration may be more readily achieved in robotic platforms, whereas conventional handheld drills would require additional sensing and signal-acquisition hardware.

### 4.5. Limitations and Future Work

Several limitations should be acknowledged. The present experiments were conducted under controlled in vitro conditions using commercially sourced dried porcine cortical bone and only a single set of drilling parameters (a constant spindle speed of 2000 rpm and a feed rate of 0.5 mm/s). Although specimen preparation and drilling conditions were standardized, the biological material itself should not be considered homogeneous. Bone density, microstructure, anatomical location, and other material properties may have varied among specimens. Moreover, because donor-specific information was unavailable, inter-animal variability and the presence of underlying skeletal pathology could not be assessed. Because drilling forces are known to be significantly influenced by rotational speed and feed rate, this single-condition testing represents a limitation of the current proof-of-concept study. Moreover, because the drilling conditions were fixed, elapsed time was inevitably correlated with drill-tip position. Therefore, although the spatially aligned signal analysis suggested that the model exploited position-related features, the present study cannot completely exclude the possibility that temporal correlations also contributed to the prediction. Future studies should deliberately disrupt this relationship by incorporating varying feed rates and rotational speeds, interrupted drilling, heterogeneous cortical thickness, and irregular drilling conditions. In addition, although MAE was used as the primary performance metric in this study, it does not distinguish the direction of prediction error and may therefore not fully reflect the safety implications of cases in which the drill tip is actually closer to or beyond the far cortex than predicted. Future studies should incorporate specimens with heterogeneous cortical thickness and varying densities, as well as diverse and dynamic speed/feed combinations, to test the model’s generalization. Drill-bit wear was not quantitatively monitored in the present study and may have influenced the force and torque signals. In addition, only a single 3.0 mm twist drill with fixed geometry was evaluated under dry drilling conditions. Clinically used drill bits vary in diameter, point angle, flute geometry, and wear state, all of which may affect drilling mechanics and sensor characteristics. Irrigation, which is frequently used during clinical drilling, may also alter the force and torque conditions. Future studies should therefore evaluate the robustness of the model across different drill-bit geometries and wear states and under drilling with irrigation.

These differences may introduce domain shift between the present experimental dataset and clinical drilling. Therefore, clinical translation will require staged validation and adaptation, including transfer learning by fine-tuning models trained on laboratory data using cadaveric and clinical data, domain adaptation techniques, multi-centre datasets collected across different instruments and institutions, and patient-specific calibration. For example, the signal recorded while drilling the near cortex of the same hole could potentially serve as an intraoperative reference for predicting breakthrough at the far cortex.

Inference latency was not measured in this study; therefore, future implementation work should evaluate real-time performance and computational efficiency, which are critical for intraoperative deployment. Finally, the current model used force and torque signals as inputs; expanding to additional sensing modalities and exploring alternative sequence-learning architectures may further enhance accuracy and adaptability to complex environments.

## 5. Conclusions

Under a controlled constant-feed condition, an LSTM model estimated the annotated drill-tip position relative to cortical breakthrough from thrust-force and torque signals with an MAE of 0.20 mm within the ±2 mm peri-breakthrough interval. Post hoc analyses suggested that the signals contained information associated with proximity to breakthrough beyond that provided by average elapsed drilling duration alone. However, time and position could not be fully separated in the present experimental design. This work represents a step toward the practical realization of Physical AI, where machines learn directly from the dynamics of their interaction with the physical world to achieve safer and more adaptive behavior.

## Figures and Tables

**Table 1:** Summary of drilling parameters across all 268 trials. Values are mean, standard deviation, minimum, quartiles, and maximum for the six parameters defined in the Methods Section.

Parameter	Mean	Std	Min	25%	Median	75%	Max
Baseline force (N)	−0.15	0.19	−2.24	−0.25	−0.15	−0.04	0.35
Start time (s)	3.18	0.87	2.00	2.54	3.02	3.64	9.01
Breakthrough time (s)	9.23	1.60	6.46	8.03	8.94	10.25	15.80
Drilling duration (s)	6.04	1.36	4.02	4.95	5.80	6.96	10.45
Max thrust force (N)	7.26	1.70	1.81	5.96	7.29	8.36	12.79
Max torque (N·m)	1.37	0.41	0.43	1.09	1.32	1.61	3.01

**Table 2:** Performance comparison of model architectures using combined thrust force and torque inputs within the ±2 mm peri-breakthrough interval.

Model	MAE (mm)	RMSE (mm)	Accuracy	Precision	Recall	F1-Score	AUC
Linear	0.538	0.669	0.895	0.910	0.872	0.868	0.982
TCN	0.404	0.527	0.895	0.929	0.857	0.880	0.966
GRU	0.327	0.361	0.944	0.946	0.950	0.943	0.998
LSTM	0.197	0.228	0.934	0.943	0.935	0.931	0.999

**Table 3:** Binary classification performance for cortical breakthrough detection within the ±2 mm peri-breakthrough window, by LSTM input configuration.

Input Configuration	Accuracy	Precision	Recall	F1-Score	AUC
Thrust force + Torque (combined)	0.934	0.943	0.935	0.931	0.999
Thrust force only	0.932	0.928	0.949	0.931	0.998
Torque only	0.939	0.936	0.951	0.937	0.999

**Table 4:** Representative sensing-based approaches to the control of bone drilling and their relation to the present study.

Approach (Representative Studies)	Signals	Method	Output	Information Provided	Setting
Force/energy-based breakthrough detection [4,5,6,20,21]	Thrust force; drilling energy	Threshold- or energy-based detection	Breakthrough event	Event-based information at or around cortical penetration	In vitro/robotic experimental
Clutch-type cranial perforators [7,8]	Mechanical resistance	Mechanical disengagement/autostop	Automatic drill disengagement	Event-triggered stopping at penetration	Clinical
ML/DL-based state or penetration classification [9,10,16]	Motor current + force; acoustic signals	Learned classifiers	Drilling state/penetration class	Discrete state or event detection	In vitro/cadaveric/clinical
Direct depth-sensing smart drill [12]	Torque + mechanical advancement	Direct depth measurement	Continuous drilling depth	Continuous direct measurement of drill advancement	In vitro bone-block model
Present study	Thrust force + spindle torque	LSTM temporal regression (data-driven)	Continuous drill-tip position relative to the far cortex	Continuous quantitative position estimate before and around breakthrough; may support anticipatory control	In vitro (porcine cortical bone)

## Data Availability

The datasets generated and analysed during the current study are available from the corresponding author on reasonable request.
